# Endodontic working length measurements of premolars and molars in high-resolution dental MRI: a clinical pilot study for assessment of reliability and accuracy

**DOI:** 10.1007/s00784-022-04636-1

**Published:** 2022-07-21

**Authors:** Mousa Zidan, Franz S. Schwindling, Alexander Juerchott, Johannes Mente, Holger Gehrig, Mathias Nittka, Zahra Hosseini, Johann M. E. Jende, Sabine Heiland, Martin Bendszus, Tim Hilgenfeld

**Affiliations:** 1 Department of Neuroradiology, Heidelberg University Hospital, Im Neuenheimer Feld 400, 69120 Heidelberg, Germany; 2 Department of Prosthodontics, Heidelberg University Hospital, Heidelberg, Germany; 3 Division of Endodontics and Dental Traumatology, Department of Conservative Dentistry, Heidelberg University Hospital, Heidelberg, Germany; 4 Magnetic Resonance, Siemens Healthcare GmbH, Erlangen, Germany; 5 Magnetic Resonance R&D Collaborations, Siemens Medical Solutions, Atlanta, GA USA; 6 Division of Experimental Radiology, Department of Neuroradiology, Heidelberg University Hospital, Heidelberg, Germany

**Keywords:** CBCT, Dmri, EAL, Endodontic working length, Magnetic resonance imaging, Working canal length

## Abstract

**Objectives:**

To prospectively assess the reliability and accuracy of high-resolution, dental MRI (dMRI) for endodontic working length (WL) measurements of premolars and molars under clinical conditions.

**Materials and methods:**

Three-Tesla dMRI was performed in 9 subjects who also had undergone cone-beam computed tomography (CBCT) (mean age: 47 ± 13.5 years). A total of 34 root canals from 12 molars (4/8, upper/lower jaw; 22 root canals) and 11 premolars (2/9 upper/lower jaw; 12 root canals) were included. CBCT and dMRI datasets were reconstructed to visualize the root canal in one single slice. Subsequently, two radiologists measured the root canal lengths in both modalities twice in blinded fashion. Reliability and accuracy for both modalities were assessed using intraclass correlation coefficients (ICCs) and Bland–Altman analysis, respectively.

**Results:**

Reliability (intra-rater I/II; inter-rater) of dental MRI measurements was excellent and comparable to CBCT for premolars (0.993/0.900; 0.958 vs. 0.993/0.956; 0.951) and for molars (0.978/0.995; 0.986 vs. 0.992/0.996; 0.989). Bland–Altman analysis revealed a mean underestimation/bias (95% confidence interval) of dMRI measurements of 0.8 (− 1.44/3.05) mm for premolars and 0.4 (− 1.55/2.39) mm for molars. In up to 59% of the cases, the accuracy of dMRI for WL measurements was within the underestimation margin of 0 to 2 mm short of the apical foramen AF.

**Conclusions:**

In vivo demonstration and measurement of WL are feasible using dMRI. The reliability of measurements is high and equivalent to CBCT. Nonetheless, due to lower spatial resolution and longer acquisition time, the accuracy of dMRI is inferior to CBCT, impeding its current use for clinical treatment planning.

**Clinical relevance:**

dMRI is a promising radiation-free imaging technique. Its reliability for endodontic working length measurements is high, but its accuracy is not satisfactory enough yet.

## Introduction

Accurate assessment of root canal morphology and preexisting pathology is crucial in treatment planning in endodontic therapy [1, 2]. Intraoral periapical radiography (PR) represents the first and easiest method of choice for visualizing periapical pathology and appropriate treatment planning [3]. However, this means three-dimensional (3D) structures are acquired in two-dimensional (2D) images and superimposed with the surrounding anatomy without any information about the spatial relationships. These limitations were recognized as early as in the 1960s [4, 5], acknowledging the restrictions of PR, in particular for the buccolingual dimension [6]. Consequently, periapical lesions are often missed on PR when compared to cone-beam computed tomography (CBCT) [7]. A new era of dento-maxillo-facial imaging emerged with the introduction of (CBCT) at the end of the last millennium [8]. Since then, CBCT has a well-established role in endodontics [9, 10].

Even though CBCT is associated with a significantly lower radiation dose compared to conventional CT-imaging [8, 11], this is substantially higher compared to conventional radiography [12]. In conjunction with the increasing application and relevance of CBCT in the management of endodontic disorders [13], this leads to an increased radiation exposure in the general population [14]. This in turn has severe effects on the young population, especially children who are more sensitive to radiation [15, 16] and therefore have a higher risk of developing cancer later on [17, 18]. Thus, the application of CBCT should be balanced out against radiation exposure. For these reasons, a 3D, non-ionizing imaging modality would be beneficial, most importantly for young patients. High-resolution three-dimensional dental MRI (dMRI) represents a new imaging modality with the potential to serve as a non-ionizing alternative to CBCT. dMRI has recently delivered promising results in endodontics [19], e.g., in detecting root fractures [20] and in detecting and differentiating periapical lesions [21, 22] as well in guided endodontics [23]. In addition, dMRI has the potential to assess pulp regeneration [24] and reperfusion [25]. These aspects have largely remained unamenable for ionizing radiation imaging modalities. Recent developments of dedicated coil and sequence techniques [26–28] have provided higher resolution and improved metal artifact–suppressed dMRI images. These improvements could allow the use of dMRI for further endodontic indications as well.

Several previous ex vivo studies have also concluded that CBCT can be used for accurate working length (WL) measurements, compared to the electronic apex locator (EAL) [29, 30]. Defining an accurate WL is an important factor for the success of root canal therapy [2, 31]. Although EAL remains the gold standard to determine accurate length measurements in most cases, using CBCT for WL measurements in patients in whom the EAL precision may be limited (e.g., due to a root perforation) provides an additional and reliable option [32]. This could therefore also be transferable to dMRI as a radiation-free diagnostic tool.

However, it remains unclear whether the new imaging capabilities of dMRI allow for a reliable and accurate analysis of the working length in clinical practice which should be comparable to CBCT. In our study, we, therefore, prospectively evaluated these parameters in vivo using dMRI and directly compared the results with the CBCT.

## Materials and methods

The study was approved by the ethics committee of the University of Heidelberg (approval number S-404/2014), and written informed consent was obtained from all participants. Overall, 9 subjects (3 males, 6 females) were prospectively enrolled in the study. Inclusion criteria were age of 18 and availability of a CBCT scan, which was performed prior to dMRI for a clinical indication (e.g., implant planning). Exclusion criteria included pregnancy, claustrophobia, and implants considered not to be safe at a field strength of 3 Tesla (e.g., cochlear implants, pacemakers, implantable defibrillators, event recorder). Exclusion criteria on tooth level were caries and/or restorations affecting or compromising the pulp, root canal, or cuspal edge (e.g., crowns, bridges, and restorations made of non-precious alloys affecting the visibility of adjacent pulps).

### Imaging

All MRI scans were obtained on a 3 Tesla MRI system (Magnetom Trio; Siemens Healthineers GmbH, Erlangen, Germany) using a dedicated 15-channel dental surface coil (Mandibula, Noras MRI products GmbH). A PD-weighted 3D MSVAT-SPACE (multiple slab acquisition with view angle tilting gradient based on a sampling perfection with application-optimized contrasts using different flip angle evolution) sequence was applied, optimized for dental MRI, as described elsewhere [27]. Sequence parameters were repetition time 1170 ms; echo time, 6.4 ms; FOV, 168 mm × 131 mm; voxel size, 0.44 mm × 0.44 mm × 0.44 mm; acquisition matrix, 384 × 300; slice oversampling 220%; slices 80; and time of acquisition, 7:45 min. All CBCT images were acquired using 3D Accuitomo 170 (J Morita; Kyoto, Japan) with the following acquisition parameters: an isotropic voxel size: 160 µm, field of view: 8 × 8 cm^2^, tube voltage: 90 kV, tube current: 7 mA, 14-bit, 360° rotation in 17 s, and 560 frames.

### Image analysis

**Fig. 1:**
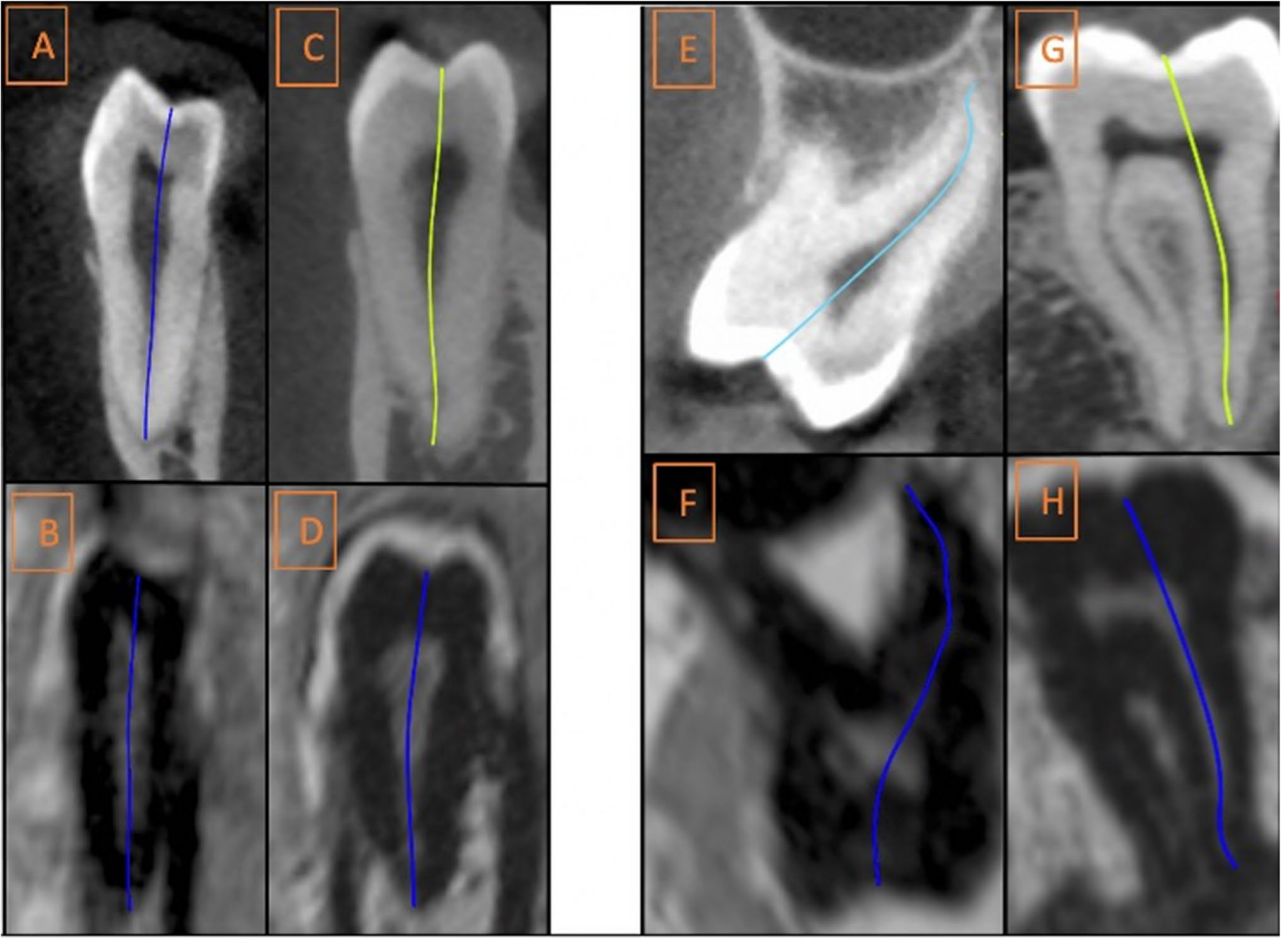
Working length measurements in CBCT and dMRI for premolars (left, **A**–**D**) and molars (right, **E**–**H**); a first left lower (**A**, **B**) and first right lower premolar (**C**, **D**); a second left upper molar (**E**, **F**) and second left lower molar (**G**, **H**)

Osirix (v. 8.5.1., Geneva, Switzerland) was used to reformat the CBCT/MRI datasets and reconstruct the slices; first, we identified the deepest intercuspal point in three planes. Then, a plane visualizing the whole length of the pulp chamber and complete root canal till the major foramen was defined. Next, images were randomized and evaluated by two radiologists (with 3 and 8 years of experience in dental imaging) twice. To avoid any influence of the reference measurements in CBCT on MRI evaluation, MRI was evaluated first. A time interval of 2 weeks separated both evaluation rounds within each modality as well as MRI and CBCT image assessment to minimize learning bias (6 weeks between first MRI read and last CBCT read). In addition, CBCT and MRI datasets were presented in different order and names in each read. Conditions between both examiners were identical. Both examiners used the same computer (MacBook Pro 15.4 inches, Model A1707, 2880-by-1800 native resolution at 220 pixels per inch) and under ACR (American College of Radiology) recommended reading room conditions [33] (diffuse ambient light of 20 lx). The radiologists could adjust the contrast/saturation of the images. The root canal length of molars/premolars was defined as the distance between the deepest intercuspal point in the projected midline of the pulp cavity and the major foramen. The measurement line followed the curvatures of the pulp cavity and was positioned in the center of the root canal until the major foramen as described before [34] (Fig. 1).

### Statistical analysis

Statistical analysis was done using SPSS 23 (SPSS Inc., Chicago, USA). To assess the reliability of both modalities, intra- and inter-rater agreement was determined by calculating intraclass correlation coefficients (ICCs) with a 95% confidence interval (*IC*). ICC values were interpreted as described before [30]. The accuracy of dMRI was analyzed with Bland–Altman plots using CBCT measurements as reference values.

## Results

The study cohort comprised 9 subjects (3 males and 6 females) who met the inclusion criteria. Mean age ± SD was 47 years ± 13.5 (median, 45; range, 29–68). A total of 34 root canals from 12 molars (4 upper jaw, 8 lower jaw; 22 root canals) and 11 premolars (2 upper jaw, 9 lower jaw; 12 root canals) were included in this study of the correlation between CBCT and dMRI measurements. Molars and premolars with apparent movement or metal artifacts during image acquisition were excluded. All root canals identified in the reference modality CBCT were included and were visible in dMRI images.

### Reliability

Intra-/inter-rater reliability of root canal length measurements for premolars was excellent for both modalities: 0.956–0.993/0.951 for CBCT and 0.900–0.993/0.958 for dMRI (Tables 1 and 2). An excellent intra-/inter-rater reliability was also noted for molars in both modalities as well with ICCs of 0.992–0.996/0.989 for CBCT and 0.978–0.995/0.986 for dMRI.

**Table 1:** Intra-rater ICC and 95% confidence interval of endodontic working length measurements in CBCT and dMRI for premolars and molars

	Rater I				Rater II			
	CBCT		dMRI		CBCT		dMRI	
	Premolar	Molar	Premolar	Molar	Premolar	Molar	Premolar	Molar
Intra-rater	**0.993** (0.978–0.998)	**0.992** (0.927–0.998)	**0.993** (0.978–0.998)	**0.978** (0.947–0.991)	**0.956** (0.928–0.973)	**0.996** (0.989–0.998)	**0.900** (0.657–0.971)	**0.995** (0.985–0.998)

**Table 2:** Inter-rater ICC and 95% confidence interval of endodontic working length measurements in CBCT and dMRI for premolars and molars

	CBCT		dMRI	
	Premolar	Molar	Premolar	Molar
Inter-rater	**0.951** (0.830–0.986)	**0.989** (0.971–0.995)	**0.958** (0.846–0.988)	**0.986** (0.966–0.994)

### Accuracy

**Fig. 2:**
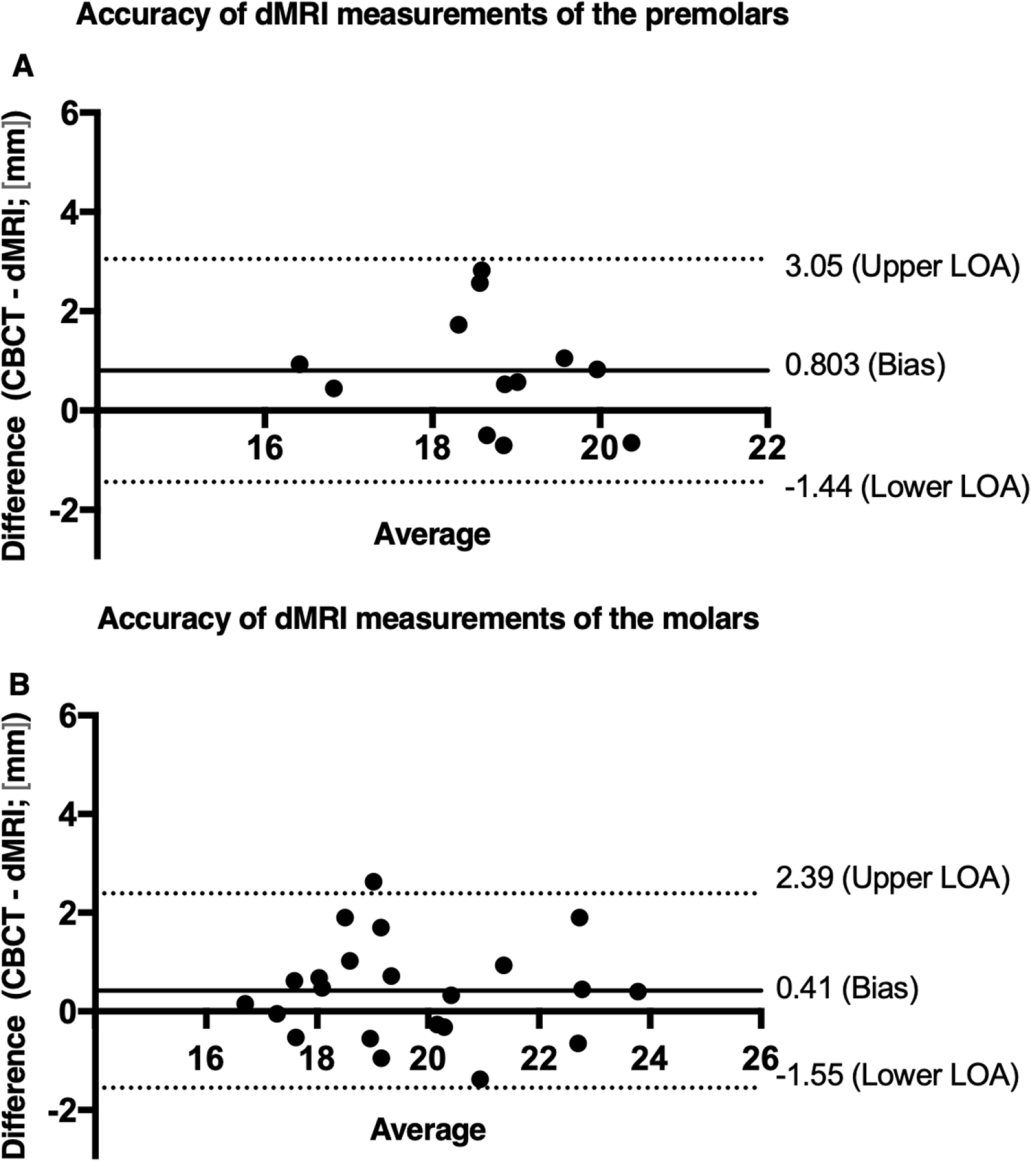
Bland–Altman plots of the mean differences between CBCT and dMRI for working length measurements for premolars (**A**) and molars (**B**) illustrating an underestimation of dMRI-derived measurements for both premolars and molars. The solid line represents the mean of all differences (bias) and the dotted lines represent the upper and lower 95% limits of agreement (LOA)

dMRI showed a tendency to underestimate the WL in 67.6% of all measurements compared to CBCT (underestimation in 75% in the premolars and 63.6% in the molars). Bland–Altman analysis uncovered a mean underestimation/bias (lower/upper limits of agreement) of 0.55 (− 1.51/2.62) mm. This translates to mean underestimation values of 0.8 (− 1.44/3.05) mm for premolars and 0.4 (− 1.55/2.39) mm for molars (Fig. 2).

The proportion of dMRI measurements within 0 to 2 mm short difference range from CBCT was 58.3% for premolars and 59% for molars. An overestimation beyond 0.5 mm of the AF was reported in 16.6/22.7% of measurements for premolars/molars. The maximum difference between dMRI and CBCT measurements was 2.83 mm in underestimations and 0.7 mm in overestimations for premolars as well as 2.63 mm in underestimations and 1.38 in overestimations for molars.

## Discussion

CBCT has become the gold standard in dental imaging, particularly in the field of endodontics, e.g., preoperative planning, to assess the extent of periapical lesions and their proximity to adjacent anatomical structures, as well as to determine the accurate number of root canals and accompanying pathologies such a periapical periodontitis and root fractures [9, 10]. However, its application is still limited due to the higher radiation dose compared to PR [35]. The higher radiation dose is associated with an increased risk of cancer later on, especially in young, radiosensitive patients [14, 18]. Consequently, a comparable, non-ionizing, 3D imaging modality like dMRI would be of high clinical value. In this study, we assessed the reliability and accuracy of dMRI for measuring the WL of premolars/molars in vivo. In terms of reliability, dMRI performed excellently and comparable to the reference imaging modality CBCT. In terms of accuracy, however, dMRI systematically underestimated WL, with a mean underestimation of 0.55 mm.

A notable benefit of our investigation is the evaluation of dMRI reliability and accuracy under in vivo conditions, thereby reproducing real-time clinical settings, where metal and motion artifacts are incorporated in the evaluation. Furthermore, all root canals identified in the reference modality CBCT were included in the study. In contrast, available information on the accuracy and reliability of CBCT in endodontic working length measurements for premolar/molars is restricted predominantly to ex vivo studies [29, 30, 36, 37]. This is problematic because ex vivo studies exclude motion artifacts, which are typical under clinical conditions with possible impact on diagnostic accuracy [38]. Liang et al. and Connert et al. included 162 and 42 extracted teeth, respectively, of which 46/42 and 12/12 were premolars/molars, respectively. Previous in vivo studies evaluating the accuracy of CBCT WL measurements only evaluated small patient cohorts and included anterior teeth only: Janner et al. (9 teeth; 6 incisors, 2 canines, 1 premolar) and Jeger et al. (40 teeth; 32 incisors, 8 canines) [34, 39]. In opposition to the previously mentioned in vivo studies, our investigation offered a comparable large sample size of 11 premolars and 12 molars. Altogether, 46 datasets and 184 root canal measurements were evaluated.

Liang et al. observed a high reliability of CBCT measurements in WL in their ex vivo study with an excellent intra-rater ICC of 0.982 [30]. Correspondingly, the reliability assessment of our CBCT measurements delivered high intra-rater ICCs as well (0.956–0.996). In a similar fashion, our analysis delivered comparably high intra-rater ICCs for dMRI in the range of 0.900–0.995. Connert et al. analyzed the repeatability in their study and reported a mean of absolute differences in CBCT measurements of 0.14 mm with a range of 0.12–0.16 mm, for all teeth included in the study. Unfortunately, the authors of that study did not report separate repeatability results for premolars and molars. In contrast, our study offered a separate repeatability assessment of the premolar/molars in both modalities, an additional strength of our investigation. We delivered a mean of absolute differences in CBCT measurements of 0.14 (0.02–0.26) mm/0.17 (0.09–0.25) mm and in dMRI measurements of 0.08 (0.02–0.15) mm/0.12 (0.1–0.13) mm for premolars/molars, respectively, illustrating an excellent and comparable repeatability in our study.

The preceding investigation of Liang et al. observed a high accuracy for CBCT-based root canal length measurements compared to EAL. Pearson correlation coefficients of 0.958 for premolars and 0.936 for molars were reported [30]; our study demonstrated a lower correlation between CBCT and dMRI measurements of 0.602 for premolars and 0.880 for molars. For assessment of absolute mean discrepancies between CBCT and EAL, Connert et al. and Liang et al. reported mean differences (range) of 0.48 mm (0.30–0.68 mm)/0.49 mm (0.30–0.66 mm) and 0.42 mm (0.03–1.12 mm)/0.51 mm (0–1.33 mm) for premolars/molars, under ex vivo conditions, respectively [29, 30]. Again, our in vivo study revealed a larger/comparable error for premolars/molars (0.8 mm /0.41 mm). The range, however, was larger for both, premolars (− 1.44 to 3.05) and molars (− 1.55 to 2.39). In another ex vivo study, Metska et al. measured 40 root canals of 33 maxillary teeth, of which 7 were molars and 11 were premolars and compared it to direct measurements [36]. The authors reported a mean underestimation of CBCT-based measurements of 0.51 ± 0.73 mm for posterior teeth (premolars and molars). In direct comparison with our dMRI results, the combined mean underestimation of 0.55 ± 1.05 mm seems comparable for both tooth types. Finally, Segato et al. compared the CBCT measurements of 30 mandibular premolars to direct measurements in another ex vivo study and reported that 73% of all measurements were within ± 0.5 mm [37]. In contrast, in our study, only 16.6% of our premolar dMRI measurement errors were within ± 0.5 mm.

In the present study, dMRI WL measurements underestimated the length in 75/63.6% of the cases for premolars/molars, respectively. Previous systematic reviews reported an improved outcome of primary root canal treatment if the root canal filling was within 2 mm short to 0 mm to the apical foramen [2]. In the present study, 58.3/59% dMRI-based measurements were within that range for premolars/molars, respectively. Moreover, in 16.6/22.7% of our dMRI measurements, there was a WL overestimation of more than 0.5 mm for premolars/molars, respectively, highlighting the lower accuracy of current dMRI technique. Nonetheless, it is essential to note that the dMRI-derived error was within the same scale as previously reported CBCT errors even though the spatial resolution as well as the acquisition time was substantially lower/longer (factor of voxel volume 21; isotropic voxel size of 0.44 mm vs. 0.16 mm from CBCT; factor of acquisition time 26; 7.45 min vs. 17.5 s).

This study has several limitations. First, CBCT and not EAL was chosen as the reference modality; even though previous studies revealed a high accuracy of CBCT-based measurements, a direct comparison with EAL or even histological results might have increased accuracy evaluation. Second, the spatial resolution of dMRI was significantly lower than CBCT, despite the application of a high-resolution dMRI setup, resulting in a lower accuracy. Third, in vivo images are prone to motion artifacts due to the longer acquisition time of dMRI compared to CBCT. That is why further developments are needed to enhance MRI image acquisition speed as well as resolution. Finally, higher costs and restricted access to MRI machines may be additional limitations for the clinical application of dMRI.

## Conclusion

This in vivo study demonstrated the feasibility of root canal length measurements for premolars and molars using high-resolution dMRI. Compared to CBCT, the intra- and inter-rater reliability of dMRI measurements was comparably high and overall excellent. In terms of accuracy of dMRI, however, root canal lengths were systematically underestimated compared to CBCT, restricting its clinical application in its current form. Further dMRI improvements resulting in higher spatial resolution and shorter acquisition times are necessary for application of dMRI in clinical routine.
